# Sero-prevalence of HBV and associated risk factors among HIV positive individuals attending ART clinic at Mekelle hospital, Tigray, Northern Ethiopia

**DOI:** 10.1186/s12981-016-0090-2

**Published:** 2016-02-04

**Authors:** Letebrhan Weldemhret, Tsehaye Asmelash, Rashmi Belodu, Dawit Gebreegziabiher

**Affiliations:** Medical Microbiology Laboratory, Mekelle Hospital, Mekelle, Ethiopia; Department of Medical Microbiology and Immunology, College of Health Sciences, Mekelle University, Mekelle, Ethiopia

**Keywords:** HBV, HBsAg sero-prevalence, Associated risk factors

## Abstract

**Background:**

Because of the shared mean of transmission, hepatitis B virus (HBV) is one of an important cause of co-morbidity and mortality in peoples living with HIV/AIDS. Hence, the aim of this study was to determine the sero-prevalence of HBV infection and associated risk factors in HIV/AIDS positive individuals attending ART clinic at Mekelle hospital, Mekelle, Northern Ethiopia.

**Methods:**

A cross sectional study was conducted from August to October 2014 in HIV/AIDS positive adult individuals. Socio-demographic data and other explanatory variables were collected from 508 study participants using pre-tested and structured questionnaire-based interviews. Serum hepatitis B surface antigen (HBsAg) was detected using commercially available rapid test and third generation enzyme-linked immunosorbent assay (ELISA). Bivariate and multivariate analysis, using SPSS V.20.0, were performed to assess the variables associated with HBV infection and *P* value less than 0.05 was considered as statistically significant.

**Results:**

A total of 508 study participants, 305 females and 203 males were included in this study with the mean (+SD) age of 37.8 + 9.6. The sero-prevalence of HBsAg was 5.9 %. Male gender (AOR = 2.6, 95 % CI 1.2–5.7), multiple sexual partners (AOR = 4.2, 95 % CI 1.3–13.1) and CD4 count <200 cells/μl (AOR = 3.5, 95 % CI 1.1–11.2) were significantly associated with HBsAg positivity.

**Conclusion:**

The prevalence of HBsAg was similar to the general population. However, HIV/AIDS positive individuals with reduced CD4 count, <200 cells/μl, showed a significant association with HBsAg seropositivity. Therefore, we recommended, all HIV/AIDS positive individuals should be screened for HBsAg during their follow for better treatment outcome and minimize risks of HBV transmission.

## Background

Human immunodeficiency virus (HIV) related mortality has been drastically reduced with expanded and wide use of anti-retroviral therapy (ART) [1, 2]. However, this improved survival of HIV/AIDS positive individuals created an enabling condition for the hepatitis B Virus (HBV) to establish chronic infection and become a major cause of co-morbidity in HIV/AIDS infected individuals [3]. More interestingly, the course of acute HBV infection may be modified in the presence of HIV/AIDS infection, with a lower incidence of icteric illness and lower rates of spontaneous clearance of HBV [4]. Therefore, the current HIV/AIDS treatment guidelines recommend the use of combination therapy, including two HBV active nucleoside analogues, for any HIV/AIDS-HBV co-infected patients initiating antiretroviral therapy (ART) [5].

Approximately two billion people have been infected with HBV and of those 400 million people developed chronic HBV infection worldwide [6]. In addition, despite the availability of effective vaccine, about one million people die annually by HBV-related disease. Approximately 4 million HIV/AIDS positive individuals are co-infected with HBV worldwide [7]. The proportion and risk factors of HBV-HIV co-infection varies widely with geographical distribution [2]. The study suggested that HBV-HIV/AIDS co-infected individuals were at increased risk of AIDS or death as compared to HIV/AIDS infected only [2, 8]. In resource limiting countries, the main route of transmission of HBV infection is vertical and/or perinatal transmission, and hence, individuals are acquired at the early age of life. Contact with infected blood or body fluids (including semen and saliva) are also common routes of transmission in developed nations [10]. However, blood and blood products are the major reservoirs for HBV infection which also shared transmission routes with HIV/AIDS [11, 12].

HIV/AIDS-HBV co-infection is common particularly in areas of endemic HBV infection [13]. Ethiopia is one of the endemic Sub-Saharan African countries for HBV. However, studies were limited to only high-risk groups, HIV/AIDS positive individuals. In Ethiopia, majorities of the previous studies were conducted in pregnant women [14] and healthy blood donors [15]. Moreover, HIV/AIDS infected individuals were treated with ART without prior screening for HBV infection. As a consequence, ART drug resistant and severe liver toxicity and cirrhosis are very high [16]. Hence, the aim of this study was to determine the sero-prevalence of HBV and associated risk factors among HIV-positive adult individuals attending ART clinic at Mekelle hospital, Tigray, Northern Ethiopia.

## Methods

### Study design and period

A cross sectional hospital based study was conducted among HIV/AIDS positive adults who visited the study site from August to October 2014.

### Study area, sample size determination and sampling method

The study was conducted at Mekelle hospital ART clinic, Mekelle, Ethiopia. The hospital is serving for about 800,500 people and giving treatment and follow-up for 4035 HIV/AIDS seropositive people. The study participants were HIV/AIDS seropositive adults who visited the ART clinic during the study period. The sample size was calculated using single population proportion formula by assuming the prevalence of HBV 6 % [15], 95 % CI and 2 % marginal error. Since the source of the study population was less than 10,000, finite population correction formula was also used to adjust the necessary sample size. Accordingly, a total of 525 study participants were enrolled including the 10 % non-response rate. The study participants were expected to represent rest of HIV/AIDS positive population in the study area because they have same socio-demographic and health service provision related matters.

### Data collection

Socio-demographic data and other explanatory variables were collected using validated and pretested questionnaire by trained nurses working at the ART clinic. Three milliliters of whole blood was also collected from each participant by venous puncture, and serum was separated. The serum was used for rapid serological HBsAg test and enzyme-linked immunosorbent assay (ELISA).

### Laboratory detection of hepatitis B virus

In-vitro serological test was performed to detect HBsAg using one step rapid immunochromatographic test (Tulip’s Instant (Tulip Diagnostics(P) Ltd. 88/89, Phase II C, Verna Ind. Est., Verna, Goa-403 722, India). The test results were interpreted and reported as positive or negative based on the manufacture’s instruction. HBsAg positive serum samples were further confirmed using 3rd generation ELISA assay at Tigray Regional Red Cross Laboratory.

### Quality control

The standard operational procedures (SOP) of pre-analytical to post analytical quality control techniques of the laboratory were strictly followed. The quality control for HBsAg test kits was monitored by using positive and negative control samples before testing the patients’ serum, and in every opening of new kits. Similarly, ELISA test results were also assured using positive and negative controls according to the manufacturer’s manual. The data quality was also maintained by training the data collectors before the actual data collection begin. Moreover, daily checking and monitoring of results were done by the principal investigators.

### Statistical analysis

Data were cleaned and entered to SPSS v. 20.0. The association between each predictor and outcome variables was determined by using binary logistic regression analysis. All predictor variables with the *P* value less than 0.2 in the bivariate analysis were entered to the multivariate model to check if the variables were associated independently [12]. Odds ratio at 95 % CI and *P* < 0.05 were used to measure the strength of the association.

The final model was built by using Enter method standard regression model building technique. Before building the final model, the multi-co-linearity effect was assessed using linear regression and the mean VIF >5 was used as cut-off point [16]. The final model was then tested for its goodness of fit by Hosmer and Lemeshow and P value > 0.05 was best fit [17]. The final multivariate model included variables that had significant association at *P* < 0.05.

### Ethical consideration

This research project was approved and ethically cleared by Ethical Review committee of College of Health Science, Mekelle University and letter of cooperation was written to the institutional review board of Tigray Regional Health Bureau for approval. Consent was also obtained from each study participant. HBsAg positive test results were communicated to the ART clinic for appropriate action and follow up. Moreover, since TDF-3TC-EFV was more effective than AZT-3TC-EFV ART combination therapy, we recommended to the regional health bureau to shift to TDF-3CT-EFV combination therapy. Our result was also presented to physicians working at the ART clinic.

## Results

### Socio-demographic characteristics

A total of 525 study participants were enrolled and of this 17 were excluded due to incomplete data. The mean(+SD) age of the study participants was 37.8 + 9.6 and most, 485 (95.5 %) were urban resident. Three hundred seventy one (73 %) of the participants were attended at least primary school. Seventy three (14.4 %) and 106 (20.9 %) were civil servants and housewives respectively (Table 1).

**Table 1:** Bivariate analysis of socio-demographic characters of HBV infection among HIV positive individuals

		HBV sero status			
Variables	Total, N = 508	Yes = 30	No = 478	COR (95 % CI)	*P* value
	Total n (%)	+ve N (%)	−ve N (%)		
Sex					
Female	305 (60)	11 (3.6)	294 (96.4)	1	
Male	203 (40)	19 (9.4)	184 (90.6)	2.8 (1.3–5.9)	0.009*
Age					
18–24	29 (5.7)	1 (3.4)	28 (96.6)	1	
25–34	152 (29.9)	7 (4.6)	145 (95.4)	1.4 (0.2–11.4)	0.782
35–44	198 (39)	18 (9.1)	180(90.9)	2.8 (0.4–21.8)	0.326
≧45	129 (25.4)	4 (3.1)	125 (96.9)	0.9 (0.1–8.3)	0.923
Residence					
Rural	23 (4.5)	2 (8.7)	21 (91.3)	1.6 (0.34–7.0)	0.564
Urban	485 (95.5)	28 (5.8)	457 (94.2)	1	
Marital status					
Single	70 (13.8)	4 (5.6)	67 (94.4)	1.8 (0.3–10.0)	0.522
Married	267 (52.6)	19 (7.1)	248 (92.8)	2.3 (0.5–10.0)	0.282
Divorced	109 (21.5)	5 (4.6)	104 (95.4)	1.4 (0.3–7.5)	0.682
Widowed	62 (12)	3 (4.9)	58 (95.1)	1	
Education					
Illiterate	135 (26.6)	10 (7.4)	125 (92.6)	1.6 (0.5–5.3)	0.428
Elementary	169 (33.3)	12 (7.1)	157 (92.9)	1.5 (0.5–5.0)	0.462
Secondary	119 (23.4)	4 (3.4)	115 (96.6)	0.7 (0.2–2.9)	0.627
Certificate& above	85 (16.7)	4 (4.7)	81 (95.3)	1	
Occupation					
Farmer	35 (6.9)	2 (5.7)	33 (94.3)	1	
Merchant	78 (15.4)	6 (7.7)	72 (92.30	1.4 (0.3–7.2)	0.706
Civil servant	73 (14.4)	7 (9.6)	66 (90.4)	1.8 (0.3–8.9)	0.500
Driver	23 (4.5)	1 (4.3)	22 (95.7)	0.8 (0.1–8.8)	0.819
House wives	106 (20.9)	2 (1.9)	104 (98.1)	0.3 (0.0–2.3)	0.260
No work	68 (13.4)	5 (7.40)	63 (92.6)	1.3 (0.2–7.1)	0.755
Other^a^	125 (24.6)	7 (5.6)	118 (94.4)	1.0 (0.2–5.0)	0.979

*COR* crude odds ratio

* Statistically significant at *P* < 0.2

^a^Self-employee, daily work, student & commercial sex workers

### HBV sero-prevalence

In this study, using the rapid immunochromatographic test, 30 (5.9 %) were positive for HBsAg and then all positive serum samples by rapid test were retested with more specific test, ELISA. Of the total HBsAg positive individuals 19 (9.4 %) were males. Regarding the marital status, 18 (6.7 %) of the HBsAg positive participants were married. Twelve (7.1 %) and 7 (6.6 %) of the participants positive for HBsAg were attended elementary school and civil servants respectively.

### Associated risk factors & clinical characteristics

In the bivariate analysis sex, multiple sexual partners, surgical history, unsafe injection, and CD4 count <200 cells/μl has shown statistically significant association with HBsAg seropositivity (Table 2). The significantly associated variables were entered into multivariate model and: male (AOR = 2.6, 95 % CI 1.2–5.7), multiple sexual partners (AOR = 4.2, 95 % CI 1.3–13.1) and CD4 count <200 cells/μl (AOR = 3.5, 95 % CI 1.1–11.2) were found significantly associated with HBsAg positivity (Table 3). Taking AZT-3TC-EFV (AOR = 2.0, 95 % CI 0.7–6.0) were showed an increased HBsAg positivity than those who were taking TDF-3TC-EFV based combined ART therapy, though the difference was not statistically significant (Table 2).

**Table 2:** Bivariate analysis of associated risk factors for HBV infection in Mekelle among HIV positive individuals

Variables	HBV sero status				
	Total = 508	Yes = 30	No = 478	COR (95 % CI)	*P* value
	Total n (%)	+ve N (%)	−ve N (%)		
Blood transfusion					
No	467 (91.9)	27 (5.8)	440 (94.2)	1	
Yes	41 (8.1)	3 (7.3)	38 (92.7)	1.3 (0.4–4.4)	0.690
Unsafe injection					
No	475 (83.5)	26 (5.5)	449 (94.5)	1	
Yes	33 (6.5)	4 (12.1)	29 (87.9)	2.4 (0.8–7.3)	0.128*
Multiple sexual partners					
No	482 (94.9)	25 (5.2)	457 (94.8)	1	
Yes	26 (5.1)	5 (19.2)	21 (80.8)	4.4 (1.5–12.5)	0.006*
Surgical history					
No	408 (80.3)	20 (4.9)	388 (95.1)	1	
Yes	100 (19.7)	10 (10)	90 (90)	2.2 (1.0–4.8)	0.058*
Tooth extraction					
No	319 (62.8)	20 (6.3)	299 (93.7)	1	
Yes	189 (37.2)	10 (5.3)	179 (94.7)	0.8 (0.4–1.8)	0.651
Abortion					
No	475 (93.5)	29 (6.1)	446 (93.9)	1	
Yes	33 (6.5)	1 (3)	32 (97.0)	0.5 (0.1–3.6)	0.478
Tattooing					
No	447 (88)	27 (6)	420 (94)	1	
Yes	61 (12)	3 (4.9)	58 (95.1)	0.8 (0.2–2.7)	0.728
Family having liver disease					
No	468 (92)	28 (6)	440 (94)	1	
Yes	40 (7.9)	2 (5)	38 (95)	0.8 (0.2–3.6)	0.800
CD4 count					
<200 cells/μl	85 (16.5)	9 (10.6)	76 (89.4.)	3.9 (1.3–12.1)	0.018*
200–350 cells/μl	118 (23.2)	7 (5.9)	111 (94.1)	2.1 (0.6–6.7)	0.221
351–499 cells/μl	135 (26.6)	9 (6.7)	126 (93.3)	2.4 (0.8–7.2)	0.133*
≧500 cells/μl	170 (33.5)	5 (2.9)	165 (97.1)	1	
ART status					
Pre ART	65 (12.8)	2 (3.1)	63 (96.9)	1	
ART	443 (87.2)	28 (6.3)	415 (93.7)	2.1 (0.5–9.1)	0.311
ART drugs					
AZT,3T, Nuv	106 (24.5)	7 (6.6)	99 (93.4)	1.2 (0.5–3.4)	0.678
AZT,3T, Evf	58 (13.4)	6 (10.3)	52 (89.7)	2.0 (0.7–6.0)	0.200
TDF,3T, Nuv	101 (23.4)	6 (5.9)	95 (94.1)	1.1 (0.4–3.2)	0.849
TDF,3T, EFV	167 (38.7)	9 (5.4)	158 (94.6)	1	

*COR* crude odds ratio

* Statistically significant at *P* < 0.2

**Table 3:** Multivariate analysis of the variables associated with HBV infection

Variables, N = 508	AOR (95 % CI)	*P* value
Sex		
Female	1	
Male	2.59 (1.179–5.688)	0.018*
Multiple sexual partners		
No	1	
Yes	4.171 (1.333–13.049)	0.014*
CD4 count		
<200 cells/μl	3.543 (1.119–11.214)	0.031*
200–350 cells/μl	1.540 (0.462–5.136)	0.483
351–499 cells/μl	2.226 (0.713–6.953)	0.168
≥500 cells/μl	1	

*AOR* Adjusted odds ratio

* Statistically significant at *P* < 0.05

## Discussion

In this study, the sero-prevalence of HBV was 5.9 % and this finding was similar with studies reported in Tigray and Amhara regions, Ethiopia (6.2 %) [18] and (5.7 %) [14] respectively. Furthermore, our finding was also similar with a study conducted in South Africa [19]. This might be due the similarity in HBV endemicity and clinical characteristic of study participants [2]. In the intermediate endemicity (2–7 %) areas, perinatal and sexual contact is the predominant routes of transmission for HBV infection [9]. On the other hand, it was relatively higher as compared with studies conducted in the United States of America (3 %) [8]. This might be due to the difference in clinical characteristics of study participants because half of the study participants in the USA study were Pre-ART (not started ART), in other words, their CD4 count were at least >200 cells/µl. However, 87 % of our participants were under ART (CD4 count <200 cells/µl) treatment [20], which an enabling condition for HBV to establish persistent infection. Other possible reasons could be the level of knowledge of HBV and HIV transmission between the communities. Other possible justification could the health services provision difference: though did not assess for vertical transmission, it could be one of the reasons for the higher prevalence in our study. On the contrary, studies conducted in Nigeria (15.4 %) [21] and China (19.4 %) [22] were significantly higher HBV prevalence and this may be due to the diagnostic tools they used, the HBV DNA RT-PCR versus HBsAg ELISA assay.

This study revealed that males were 2.59 times more likely to expose with HBV than females. Similarly, history of having multiple sexual partners was significantly more frequent among males (6.9 %) than females (3.9 %) (Table 3).The possible explanation could be, in developing countries, especially in the rural and semi-urban communities, because of their job nature, males travel more frequently than females. This was comparable with studies conducted in Gondar teaching hospital, Ethiopia, Pasteur institute, Morocco and Pakistani Punjab [14, 22–25].

Though the difference was not statistically significant, the mean CD4 cell count (Mean + SD) for HBV-HIV/AIDS co-infection (320 + 126 cells/μl) was lower than HIV/AIDS alone (402 + 201 cells/μl). The reason for is; at very lower CD4 count (200 cells/µl), the course of HBV infection may be different from the immune-competent individuals, meaning the primary infection will be a mild liver disease with a lower incidence of icteric and lower rates of spontaneous clearance of HBV. However, this is enabling condition for HBV to establish chronic infection [4, 23–26]. This finding was consistent with studies conducted in Gondar, Ethiopia [14] and Nigeria [27].

Furthermore, significantly higher HBsAg seropositivity was observed among individuals with CD4 count <200 cells/ul (AOR = 3.543, 95 % CI 1.119–11.214) than individuals with CD4 count ≥500 cells/μl (Table 3). In this study, we compared the HBsAg seroconversion rate between study participants who were taking AZT-3TC-EFV and TDF-3TC-EFV combined ART therapy and statistically insignificant higher (Adjusted *P* = 0.2) HBsAg seropositivity was observed in AZT-3TC-EFV treatment follow up attendants. This can be explained by efficacy difference between the two treatment regimen; where TDF-3TC-EFV, a nucleotide analogue which inhibited the polymerase and viral replication, has more efficient than lamivudine (3TC) and Zidovudine(AZT) in the treatment of both HIV/AIDS and HBV [28].This was similar with a study conducted in Kenya [29]. Similarly, other studies also reported that TDF-3TC-EFV combined ART therapy significantly reduced the level of HBV DNA level in the patient’s serum [28, 30]. another interesting result from the African Temprano Trial, presented at the conference on Retroviruses and Opportunistic Infection (CROI 2015), showed that starting HIV/AIDS treatment (TDF was one of the drugs they used) at a CD4 cell count above 500 reduced the risk of serious illness and death by 44 % when compared to starting treatment according to WHO guidelines [31].

Socio-demographic characteristics like age, residence, marital status, education and occupation were not significantly associated to HBsAg positivity. Likewise, of the risk factors history of blood transfusion, unsafe injection, tooth extraction, history of surgery, catheterization, abortion, tattooing and having a history of family liver disease did not show statistically significant association with HBV infection and this was consistent with studies done in Goba, Ethiopia [32].

## Conclusion

HBsAg seropositivity among individuals with CD4 count <200 cells/µl is significantly higher than with CD4 count >200 cells/µl. HBsAg seropositivity was also higher among the AZT-3TC-EFV than TDF-3TC-EFV combination ART therapy; could be justified by the TDF, a nucleotide analogue, which efficiently inhibit the viral polymerase and replication cycle than the AZT. Regarding sex distribution, males were frequently infected with HBV than females. Furthermore, in this study we were also observed a greater HBV prevalence among individuals having a history of multiple sexual partners. Therefore, this highlights the need to strengthen and integrate HBV screening and treatment with the existing HIV/AIDS Prevention and Control Policies.

## Abbreviations

AIDS: acquired immunodeficiency syndrome
ALT: alanine transaminase
ART: anti-retroviral therapy
AST: aspartate transaminase
CD: cluster of differentiation
CD4: CD4 + T cells (T lymphocyte bearing CD4 receptor)
DNA: deoxyribonucleic acid
ELISA: enzyme-linked immunosorbent assay
CHB: chronic hepatitis B
HAART: highly active antiretroviral therapy
HBcAg: hepatitis B core antigen
HBeAg: hepatitis B e antigen
HBsAg: hepatitis B surface antigen
HBV: hepatitis B virus
HCC: hepatocellular carcinoma
HIV: human immunodeficiency virus
mRNA: messenger ribonucleic acid
PCR: polymerase chain reaction
TDF: tenofovir
USAID: United States Agency for International Development
WHO: World Health Organization
3TC: lamivudine
AZT: zudovidine
Nuv: nuvirapine
EFV: efavirinaze
VIF: variance inflation factor
